# 3 T cardiac magnetic resonance performs well as the primary scanner in a clinical setting: our initial experience at a tertiary care center

**DOI:** 10.1186/1532-429X-11-S1-O101

**Published:** 2009-01-28

**Authors:** Mahadevan Rajaram, Luciana F Seabra, Shuaib M Abdullah, Sanjeev A Francis, Sofia C Masri, Renate Jerecic, Michael Jerosch-Herold, Raymond Y Kwong

**Affiliations:** 1 Brigham and Women's Hospital, Boston, MA USA; 2 Siemens Medical Solutions, Chicago, IL USA

**Keywords:** Excellent Image Quality, Parallel Imaging Technique, Banding Artifact, Cine SSFP, High Acceleration Factor

## Introduction

Despite the advantage of increased signal-noise-ratio, skepticism exists regarding the use of 3 T as the primary scanner for routine clinical CMR examination due to potential for gating difficulties related to the increased magnetohydrodynamic effect, off-resonance artifacts, and patient heating. We quantified the diagnostic potential and artifacts based on our experience of the first 4 months of routine clinical 3 T CMR exams in a tertiary clinical center.

## Purpose

To test the hypothesis that 3 T MRI is practical in serving a busy clinical CMR service as the primary routine cardiac scanner.

## Methods

Two-hundred and eighty patients were referred for CMR for a broad range of clinical indications over a 4-month period and underwent a 3 T cardiac MRI scan (MAGNETOM Tim Trio, Siemens, Germany). Three experienced readers quantified total scan time, troubleshooting time for 3 T-related off-resonance artifacts, image quality, and artifacts in all pulse sequences performed. Image quality was graded per accepted criteria (1-Non diagnostic, 2-diagnosis suspected but not established with severe blurring, 3-definite diagnosis despite moderate blurring, 4-definite diagnosis with only mild blurring, 5-definite diagnosis without visible blurring). Artifacts severity was graded in a 5-point scale (1-No artifacts, 2-minimal artifacts, good diagnostic quality images, 3-moderate artifact and diagnosis established, 4-considerable artifacts, diagnosis suspected but not established, 5 – severe artifacts, non diagnostic images). Excellent image quality was classified as a score ≥ 4 and minimal or no artifact was classified as an artifact score of ≤ 2. Forty-six 1.5 T CMR studies performed at the same study period with a matched spread of indications were randomly selected as a control group for comparison.

## Results

**Figure 1:**
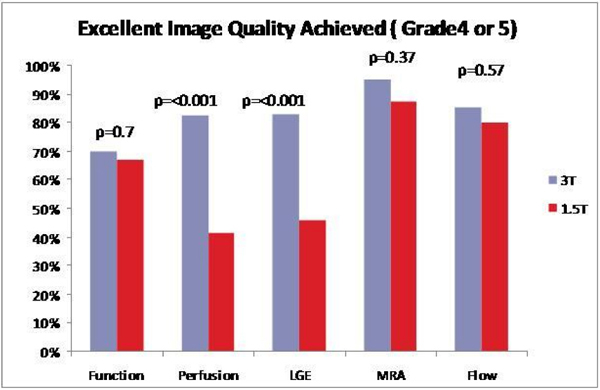
**Excellent image quality achieved (grade 4 or 5)**.

On average, 2.8 minutes (5% of total scan time) were spent to eliminate off-resonance banding artifacts in 3 T. This time is made up by more aggressive accelerated parallel imaging technique. As a result, average total scan time using 3 T was not different from 1.5 T (54 ± 14 vs. 54 ± 12 minutes, P = 0.47). No patients failed to complete the study due to SAR limit. There were no complications during any of the 1.5 T or 3 T CMR studies. A significantly higher proportion of perfusion images were graded as being of excellent quality on 3 T when compared to 1.5 T (82.4% vs. 41.4%, p < 0.0001) (Figure 1). A significantly higher number of perfusion images also had minimal or no artifact on 3 T when compared to 1.5 T (93.7% vs. 72.4%, p = 0.0016). When LGE images were analyzed, a significantly higher proportion of images on 3 T were graded as being excellent (82.6% vs. 46.2%, p < 0.0001) and the proportion of LGE images having minimal or no artifact was also significantly higher on 3 T (83.0% vs. 56.4%, p = 0.0042). The number of Cine SSFP, pulmonary vein MRA, and phase contrast images that were graded as being of excellent quality or with no or minimal artifact did not differ between 3 T and 1.5 T.

## Conclusion

3 T cardiac MRI performs well serving as the primary scanner in a busy CMR service with comparable scan times to 1.5 T cardiac MRI. 3 T has improved image quality and fewer artifacts especially for applications like perfusion and LGE which benefit from the increase in T1 times at 3 T. The high SNR leaves additional room to also decrease the overall scan time in the future using higher acceleration factors for parallel imaging techniques without sacrificing diagnostic image quality.

